# A randomized controlled trial to examine the effectiveness of the Dutch version of the Program for the Education and Enrichment of Relational Skills (PEERS®)

**DOI:** 10.1186/s12888-022-03913-3

**Published:** 2022-04-22

**Authors:** Sakinah Idris, Bjorn Jaime van Pelt, Gabrine Jagersma, Jorieke Duvekot, Athanasios Maras, Jan van der Ende, Neeltje van Haren, Kirstin Greaves-Lord

**Affiliations:** 1 Department of Child and Adolescent Psychiatry/Psychology, Erasmus MC-Sophia Children’s Hospital, Wytemaweg 8, 3015 CN Rotterdam, The Netherlands; 2 Department of Psychiatry, Faculty of Medicine, Universiti Teknologi MARA, 68100 Batu Caves, Selangor, Malaysia; 3 Yulius Organization for Mental Health, Burg. De Raadtsingel 93c, 3311 JG Dordrecht, The Netherlands; 4 GGZ Rivierduinen Children and Youth, Institute for Mental Health, Leiden, The Netherlands; 5 Autism Team Northern-Netherlands, Jonx Department of (Youth) Mental Health and Autism of Lentis Psychiatric Institute, Groningen, The Netherlands; 6 Department of Psychology, Clinical Psychology and Experimental Psychopathology Unit, University of Groningen, Groningen, The Netherlands

**Keywords:** Autism Spectrum Disorder, Adolescence, PEERS®, Social skills intervention, Behavioral observation

## Abstract

**Background:**

This study examines the effectiveness of the culturally adapted Dutch version of The Program for the Education and Enrichment of Relational Skills (PEERS®), utilizing a randomized control trial (RCT) with an active treatment control condition.

**Methods:**

106 adolescents with ASD, aged 12–18 years, were randomly assigned to one of two group interventions: the experimental condition (PEERS®; *n* = 54) or the active treatment control condition (Regulation, Organization and Autonomy Didactics; ROAD; *n* = 52). Effects of interventions on social skills were primarily assessed using an observational measure (CASS – Contextual Assessment Social Skills). Secondary indices of social skills were self, parent and teacher reported questionnaire data (i.e., Social Responsiveness Scale; SRS, and Social Skills Improvement System; SSIS). Treatment satisfaction was also obtained from adolescents and their parents.

**Results:**

Results on the observational measure of social skills revealed improvements in positive affect, overall quality of rapport, as well as starting and ending a conversation, irrespective of condition. Compared to ROAD, PEERS® participants showed increased overall self-reported social skills (SSIS). Parent reports showed decreased overall social skill impairment (SRS) as well as improved social communication (SSIS subscale), with significantly more progress in the PEERS® group. Furthermore, parents of adolescents in the PEERS® group were significantly more satisfied with the intervention (M = 8.20, SD = 1.46) than parents of adolescents in the ROAD group (M = 7.52, SD = 1.45). The self-reported treatment satisfaction of adolescents did not differ between conditions. Teacher data showed decreased social skill impairment as measured with the SRS, irrespective of condition.

**Conclusions:**

This study reveals promising indications that the Dutch version of PEERS® enhances social skills in adolescents with ASD. Yet, further research is needed into how effectiveness can be optimized.

**Trial registration:**

Dutch trail register NTR6255 (NL6117) 08/02/2017 https://www.trialregister.nl/trial/6117

**Supplementary Information:**

The online version contains supplementary material available at 10.1186/s12888-022-03913-3.

## Background

Adequate social skills are essential to develop meaningful relationships. Being able to initiate and maintain conversations increases the likelihood of peer-acceptance and long-term interpersonal relationships [1]. Limitations in social communication and subsequent difficulties in relating to others are major challenges for adolescents with autism spectrum disorder (ASD). Adolescents with ASD often have few friends, and experience increased peer rejection, resulting in greater functional impairment and poorer quality of life (e.g., difficulties achieving personal potential and positive social involvement) [2, 3]. For these reasons, they are often referred to social skills training. Yet, evidence-based social skills interventions for adolescents with ASD are only available in a few countries. Cultural adaptation of evidence-based interventions is therefore needed. Cultural adaption entails that the intervention it is tailored to a cultures’ (1) language, (2) customs, and (3) care/community system [4, 5]. First, *language* refers to patients' preferred language, such as the particular language/slang used by adolescents. Second, *customs* refer to the customs of a particular society (e.g., common activities performed with friends or effective responses to teasing). Third, *care/community system* refers to the way in which formal care-settings and informal peer group activities are organized in a community. Cultural adaptations of existing, well-validated interventions that are systematically documented and tested, can update practice and improve broader implementation [5].

A growing body of research shows efforts to culturally adapt social skills interventions for individuals with ASD [1]. An originally US evidence-based social skills intervention that has been culturally adapted and validated in several cultures such as Korea [6], Israel [7], Hong Kong [8] and Japan [9], is the Program for the Education and Enrichment of Relation Skills—parent assisted (PEERS®; [10, 11]). The PEERS® intervention teaches ecologically valid social skills to adolescents with ASD, while their parents are simultaneously being trained to become their social coaches, to help execute and maintain the learned social skills of their child [10].

In this article, we aim to extend the cross-cultural research on the PEERS® intervention by testing the effectiveness of the Dutch cultural adaptation. PEERS® was first shown to be an effective social skills group intervention for adolescents with ASD in the United States of America [10, 12–14]. Post-intervention, US participants in the treatment group were found to have improved their social skills knowledge, overall social skills, and social engagement, while autistic mannerisms decreased as compared to the control group. Improvements were maintained at long-term follow-up, 1 to 5 years post-treatment [14]. In a growing number of other countries (such as Israel, Japan, Korea, and Hong-Kong) cultural adaptations of PEERS® also appear to be effective in improving the social skills of adolescents with ASD [6–9]. The cultural adaptations have a few concrete aspects in common, such as the culture specific examples of social groups, activities, social networking sites, jokes as well as wording for teasing comebacks (for an overview of these previous studies, please see Additional file 1).

In the Netherlands, the culturally adapted version of PEERS® has yielded promising preliminary results in a pilot-study [15, 16]. Apart from the linguistic adaptation, minor adjustments were made for the Dutch version, including culture specific examples of social groups, activities, social networking sites, jokes as well as wording for teasing comebacks. The most striking cultural adaptation was the adaptation of the ecologically valid way of responding to gossip, acting un-impressed/underwhelmed (Dutch version) rather than amazed (US version) [16]. Encouraged by the pilot results, we initiated a Randomized Controlled Trial (RCT) to more rigorously examine the effectiveness of the Dutch version of PEERS® among cognitively able adolescents with ASD.

The overall objective of the current study was to examine the effectiveness of the Dutch version of PEERS® in improving social skills among adolescents with ASD. We intended to replicate and extend earlier findings in other cultures, by using a rigorous RCT design. Based on previous findings (see additional file 1), the current study sought to test whether (1) adolescents with ASD in the PEERS® condition exhibit significantly better improvements in social skills (as assessed with observations and questionnaire data) compared to those in an active control condition; (2) adolescents and parent’s satisfaction in the PEERS® condition are significantly higher than in an active control condition.

## Methods

### Overall RCT design

Our RCT differs from previous RCTs regarding four main methodological aspects:

First, we implemented an active control condition instead of a waiting list condition. The use of an active control condition, in which a similarly sized standardized protocol is administered to those participants, ensures that the outcome of the PEERS® condition can be attributed to this experimental intervention and not to non-specific treatment components [17].

Second, next to parent and teacher reports, we added self-reports, using a validated and well-established self-report questionnaire of social skills (SSIS), to obtain information on potential self-perceived improvements in social skills by adolescents with ASD themselves.

Third, we used a behavioral observation as our primary objective intervention outcome measure, i.e., objective meaning not being biased by the perspectives of any of the stakeholders, namely the Contextual Assessment of Social Skills (CASS; [18]).

Finally, unlike previous RCTs, we acquired data on social validity. Social validity refers to how well the intervention procedures are accepted and how satisfied participants are with the intervention.

### Recruitment and inclusion criteria

Eligible participants were recruited via three routes. First, participants with ASD were directly referred by psychologists/psychiatrists/pedagogues (who were independent of the PEERS® or ROAD intervention, i.e., not providing it themselves) from three participating mental healthcare institutions in the South-western and North-eastern provinces of the Netherlands. Second, the potential participants from other mental healthcare institutions with specialised ASD in- and outpatient care in the Netherlands were referred to either one of the three participating centres. Third, adolescents and their parents applied for participation in the study themselves after reading the information on websites, leaflets/posters or on social media or via referral by their general practitioner. After referral, adolescents and their parents were contacted by phone to inform them about the study and detailed information was send to them. After a week, eligible participants were contacted again to see if they had additional questions after reading the information, and to check in- and exclusion criteria.

Inclusion criteria were (1) diagnosis of ASD (DSM IV or DSM V); (2) aged between 12 and 18 years old; (3) a total and verbal IQ score ≥ 70 (assessed with the WISC-IV or WASI); (4) motivation of the adolescent to—together with his/her parents—participate in the study, and (5) enrollment of the adolescent in secondary education. Participants were excluded if they were identified with either one of the following: (1) a history of a severe mental illness except ASD (e.g., schizophrenia, bipolar disorder, or other types of psychotic disorders), or (2) any clinically relevant visual, hearing, or physical impairments that prohibited participation in the intervention/study.

Subsequently, after agreeing to participate, adolescents and their parent(s) were invited for a 60-min intake interview. The goal of the intake was to assess their motivation, to check whether their treatment needs were in line with the interventions, to further inform them about the procedures, and examine their understanding of the information that had been provided. Assessment of motivation was done with an eleven-point Likert-scale question (0 not motivated at all /10 – completely motivated) for both interventions, and additional/clarifying motivation questions during the interviews with adolescents and parents separately. During the intake, the adolescent and his/her parents gave permission to contact their teacher. Written informed consent was obtained from all adolescents and their parents during the intake interview. The consent from teachers was obtained once the teacher agreed to participate in this study.

This study adhered to all guidelines under the ethics approval of Erasmus Medical Center, Rotterdam (MEC-2016–357).

### Sample size calculation

The sample size for this study was determined by power calculations using G*Power 3.1 [19] based on the mean and standard deviation of post-treatment results of the primary outcome measure, the CASS from a previous study [13]. We needed at least 64 participants in each condition (total *n* = 128) to detect a difference of a moderate effect size (d = 0.50) between the experimental condition and the control condition with a power of 0.80 and an alpha of 0.05. Thus, we aimed for a total of 150 participants, taking into account a non-response/drop-out rate of ~ 15%. This percentage was based on a previous RCT in this population at the same centres [20].

### Participants and assessments

Adolescents with ASD and their parents were recruited for participation in the study between January 2017 and October 2019. All participants were between 12 and 18 years old and had an intelligence quotient (IQ) of > 70. In addition, all participants were previously diagnosed with ASD following DSM-IV-TR or DSM 5 criteria by a multidisciplinary team of licensed psychiatrists and psychologists [21].

The adolescents and their parents were assessed at baseline (T1) through questionnaires. The adolescents were also assessed using a behavioral assessment; the Contextual Assessment of Social Skills (CASS). Teachers, blinded for treatment condition, completed a set of questionnaires online. After the baseline assessment, participants were randomly assigned to one of the two conditions: the experimental treatment condition group (PEERS®) or the active control condition group (the Regulation, Organization and Autonomy Didactics intervention, ROAD). Allocation to conditions was determined using a computer-generated blocked randomization procedure, generated by staff members who were not involved in the study. Then, the research assistant enrolled and assigned the participants to the interventions. Researchers who were involved in the analyses were blind to the treatment allocation. Participants were instructed not to tell their teacher which intervention they received.

Data from adolescents, parents and teachers were collected at four time-points: T1 (baseline, 1 week prior to start of the intervention), T2 (intermediate: halfway during the intervention, week 7), T3 (post, at the end of the intervention, week 14) and T4 (follow-up: 14 weeks after the end of the intervention). The CASS was administered at T1, T3 and T4, because improvement in social skills were not yet expected after 7 weeks of training (at T2). We did not expect that the taught social skills knowledge would already be consolidated and transferred to observable behaviour at that point. Adolescents and parents completed all questionnaires on paper, except for T2, when questionnaires were completed online. The researchers were present during T1, T3 and T4 assessments to coordinate the paper and pencil filling out of the questionnaires. The adolescents and parents however completed the questionnaires themselves, this was not done by the clinicians. These assessments took place at the participating mental health care institutions. For the IQ assessment, a trained researcher administered and coded the WISC/WASI. Ratings by teachers were collected online at all time-points. The study design is described in more detail in van Pelt et al. [22].

### Interventions

The treatment condition group followed the PEERS® intervention that consists of fourteen 90-min sessions, delivered once a week over the course of 14-weeks. The program is a manualized social skills intervention that targets the improvement of ecologically valid social skills among adolescents with ASD, involving parents by training them how to best socially coach their child [10, 11, 23]. The program addresses elements of social functioning for adolescents, such as two-way conversational skills, making friends, using appropriate humor, handling rejection, bullying or rumors. The social skills are taught using cognitive-behavioral treatment techniques (i.e., psychoeducation, Socratic questioning, role play demonstrations, rehearsal exercises, homework assignments and homework review). The homework assignments require the adolescent to rehearse the newly learned skills with peers in daily life to enhance generalization of the skills to other settings. Parallel parent sessions focus on discussing homework and how parents can support and supervise the repetition and rehearsal of the newly learned skills of their child. Parents act as a social coach for the implementation of these skills in natural settings. In the Netherlands, the PEERS® manual was translated and adapted by a team of 15 mental health care professionals [15, 16].

Participants in the active control condition group received the ROAD (Regulation, Organization and Autonomy Didactics) intervention, an integration, adaptation and extension of two, in the Netherlands used, psycho-education training programs for adolescents with neuropsychiatric disorders: *Tackling Teenage* [20] and *Power Coaching* [24]. ROAD also consists of fourteen 90-min sessions, delivered once a week over the course of 14-weeks. The ROAD group psycho-education program covers a broad range of teenage relevant themes, such as self-knowledge, self-acceptance, self-management, planning/organisation, and discusses intimate (taboo) topics such as dealing with negative emotions (e.g., anger against self and others) and psychosexual exploration (including gender identity). ROAD has the purpose of generally helping adolescents with ASD to improve their daily functioning and in turn their overall wellbeing and quality of life. ROAD was not specifically developed to enhance and train social skills, and therefore is suitable as an active control intervention.

Groups in both interventions consisted of 4–10 adolescents, under the supervision of at least one certified and experienced clinician and accompanied by another clinician or coach (e.g., psychology student with Bachelor level). Each session lasted 90 min. The outlines of the PEERS® and ROAD sessions are constructed similarly, i.e., homework review, didactic lesson, practice (PEERS®) or discussing didactic lesson (ROAD), and homework assignments for the following week. In the PEERS® intervention, parents are involved in 14 parallel social coaching sessions. In the ROAD intervention, parents only receive an outline of the didactics, homework assignments and a short summary report after each session by e-mail.

### Measures

In this paper, we focus on the primary outcome domain of social skills. As a primary index to assess social skills, we selected the CASS, based on its objectiveness and face validity [22]. In addition, we describe results on two secondary measurement tools, i.e., parent, self, and teacher reports on other indices of ‘social skills’ (i.e., the SRS-2 and SSIS).

#### Diagnostic and demographic measures

Several diagnostic and demographic measures were obtained for descriptive purposes and were considered as potential co-variates:

- *1. ASD symptom severity* ASD symptom severity was determined using the Autism Diagnostic Observation Schedule Second Version (ADOS-2; [25]). The ADOS-2 has good test–retest reliability (0.82) and inter-rater reliability (0.92). The ADOS-2 consists of four modules, each designed to be administered to individuals according to their level of expressive language. Module 3 and 4 were used in the current study, based on the developmental age as well as the language abilities of the participating adolescents. The total scores were computed using the calibrated severity score (CSS) ranging from 1–10. The “Autism Spectrum Disorder” classification includes scores in the range from 4–10 and the “Non-spectrum” classification includes scores in the range from 1–3. If the ADOS-2 had been administered by a trained and licensed clinician as part of routine clinical procedure in the past 5 years, then those scores were distracted from the patients file with permission from the parents. If the ADOS-2 was not available from the record, a trained and licensed researcher administered the ADOS-2. Meeting the ADOS dichotomous cut-off was not considered a prerequisite for participation, rather the CSS was used as a descriptive index of ASD severity. Please note here that the ADOS-2 was assessed to obtain a more objective index of ASD severity, but it was not considered to be needed as a ‘confirmation’ of clinical diagnoses. Although the ADOS-2 is a well validated assessment tool and is considered as an important part of the entire diagnostic process, the best-estimate clinical consensus DSM classification based on all available information (such as ADOS-2, but also developmental anamneses, neuropsychological testing etc.) is considered the gold standard for diagnosis [21] and was therefore used to determine eligibility.
- *2. Cognitive ability (Intelligence Quotient)* If available, information on the Intelligence Quotient (IQ) was extracted from the patient file with the permission from the parents. If the information was more than 5 years old, the Wechsler Intelligence Scale for Children (WISC-IV [26];) or Wechsler Abbreviated Scales of Intelligence (WASI; [27]) was assessed by a trained researcher in this study.
- *3. Demographic and previous/concurrent treatment information*. Information on demographics (i.e., age and biological sex of the adolescent and parent) and previous/concurrent treatment (i.e., current psychotropic medication and pre-occurring social skills training) was obtained through a questionnaire that was filled out by the parent at pre-assessment (T1).

### Primary outcome – Observational measure

- *1. The Contextual Assessment of Social Skills (CASS)* is an observational measure of social functioning and conversational skills developed for cognitively able adolescents and young adults with ASD. During the CASS, participants experience a conversation with a confederate (i.e., an unfamiliar, opposite sex, similarly aged peers without ASD) for three minutes. The assessment starts when the test leader reads the instruction to the participant, outside of the room where the confederate is already seated. The participants are asked to fill out the level of confidence they feel before they enter the room (on a scale from 1 – 10, 10 being totally confident). Then, the participant enters the room. The participant is instructed to start the conversation, while the confederate is instructed to wait to ensure that the participant can demonstrate initiation skills. After 3 min, the test leader knocks on the door as a sign to end the conversation. Subsequently, the participant and the confederate are asked to complete a brief questionnaire about how they experienced the conversation (Conversation Rating Scale; CRS, see below), that comes with the CASS.
  During the conversation, confederates demonstrate warm, friendly, and interested behavior. Confederates are also instructed to display appropriate nonverbal cues, such as smiling, an appropriate level of eye contact, an open posture, and appropriate gestures. They are instructed to be supportive of the conversation and leave room for the participant to mainly lead the conversation. For more details, please see van Pelt et al. [22].
  The conversation was videotaped for later coding. Coding included participant and confederate’s verbal and non-verbal behaviors, across the original nine CASS rating domains; (1) Asking Questions, (2) Topic Changes, (3) Vocal Expressiveness, (4) Gestures, (5) Positive Affect, (6) Kinesic Arousal (i.e., signs of physical arousal, such as fidgeting or repetitively moving body parts), (7) Social Anxiety, (8) Overall Involvement/ Interest in the Conversation, and (9) Overall Quality of Rapport. The first two items are rated as frequency counts. The other seven items are rated on a Likert scale ranging from 1 = low to 7 = high. Scores below 6 indicate social skills deficits [18, 28]. Four new additional domains were also coded. Within domain 1, we separated between (1a) Initiating and (1b) Follow-up Questions, as these are specifically instructed within PEERS®. The other three domains are binary items (yes/no) i.e., Domain 0; Starting the Conversation, Domain 10; Initiating the end of the conversation, and Domain 11; Giving a reason to end the conversation. These additional domains were introduced to fit more closely to the PEERS® learning objectives. In line with the previous research [13], only the seven original Likert scale rating domains were used to compute the CASS Total score, because the total of the *nine* original rating domains had a lower internal consistency (Cronbach alpha = 0.84) than the CASS Total score consisting of the sum of the *seven* original rating domains of the Dutch CASS (Cronbach alpha = 0.86). Moreover, the frequency count items may conflict with the specific skills and social conventions taught in PEERS® (e.g. the rule “don’t be an interviewer”, that conflicts with counting the number of questions asked as an index of social skills, since asking too many questions might actually be considered too interruptive/dominant, [13, 23]. Therefore, these two frequency items were not integrated in the CASS Total score, but the results on these items were analyzed separately. Also, the results on the newly developed additional four domains were analyzed separately.
  Fifteen undergraduate students were trained to score the CASS, by coding six training videos (three original US videos and three Dutch videos). Because this study used a fixed number of coders, we compared the scores of each coder against a gold standard (i.e., the codings by KGL who received training by the original CASS developer Ratto). The coders achieved at least 70% overall agreement with this author's code and were then considered as an eligible coder. The coders were blinded to the time points and condition to minimize bias. The videos were also used for coding the behaviors of the confederates, since statistically significant differences in CASS scores amongst participants were found to be related to the CASS scores of confederates [13]. Therefore, confederate CASS scores were considered as a potential covariate in the current study.
- *2. The Conversation Rating Scale (CRS)* is a report on the social behaviors of the confederate as perceived by the participant, and vice versa, during the CASS conversation [28]. It consists of five items which cover perceived interest, friendliness, conversational flow, perceived boredom, and sense of distance rated on 7-point Likert scale ranging from 1 (strongly disagree) to 7 (strongly agree). Note that also confederates scored the CRS about the participant. Two items about self-confidence (before and after the conversation) and three items about the perspective on the conversational partners’ interest and current/future engagement were added, to align more closely to the learning goals of the PEERS® program (i.e., answering the perspective taking questions that go with this program).

Before and following each conversation, participants and confederates completed the Conversation Rating Scale (CRS) items. Following the procedure by Ratto et al. [18], the original 5-item scores were summed (with the last two items reversely scored) to generate a total score of perceived conversational interest. The total score ranged from 5 to 35, higher score indicating higher levels of perceived interest/engagement of the other person (e.g., perceived interest of the confederate as reported by the participant, or perceived interest of the participant as reported by the confederate). Internal consistency for this CRS total score in our sample was high (Cronbach’ alpha = 0.75). The newly developed additional five items were analyzed separately. The self-confidence items were analyzed individually. The three items on perspective taking were summed (scores ranging from 3 to 21, higher scores indicating higher confidence in the reciprocated appreciation of the conversation).

### Secondary Parent-, Teacher- and Self report Measures

- *1. Social Skills Improvement System (SSIS)* is a questionnaire for the assessment of social skills at home, in the classroom and in interactions with peers [29]. The SSIS was administered to adolescents, parents, and teachers (each version has 46 items). The social skills subscales were used, which include communication, assertion, empathy, engagement, and self-control. It takes 15 min to complete and has shown to be sensitive to change in social skills among high functioning adolescents with ASD participating in PEERS® [30]. The internal consistency for the total SSIS parent, adolescent, and teacher were high in the current sample; Cronbach alpha = 0.78, 0.85, and 0.80, respectively.
- *2. Social Responsiveness Scale-version 2 (SRS-2)* is a 65-item questionnaire with a 4-point scale from 0 (not true) to 3 (almost always true), with the total score ranging from 0 to 195. It measures the severity of social impairment related to ASD [31] and was completed by parents and teachers. The SRS-2 [32] is used for children aged 4–18 years and has an acceptable model fit with the two-factor structure of ASD, as conceptualized in the DSM-5 [33]. It provides information for specific subscales (i.e., social awareness, social cognition, social communication, social motivation, and autistic mannerisms). Consistent with the validation studies in other countries, the Dutch version of the parent report SRS-2 demonstrated high internal consistency (Cronbach alpha ranged from 0.92 to 0.95, good convergent validity; *r* = 0.63 with the ADI-R) and was able to differentiate between children with ASD versus those from the general population [34]. In the current study, the Cronbach alpha of the SRS-2 subscales ranged from 0.31—0.84, with a Cronbach alpha of 0.77 for the Social Communication Index (i.e., the sum of the social awareness, social cognition, social communication, and social motivation subscales).

### Process evaluation variables

Information on the quality of the delivery of the interventions was obtained using the following measures. These measures were used for descriptive purposes and considered as potential covariates:

- *1. Treatment satisfaction* was measured using a self-developed treatment satisfaction questionnaire, that was administered to adolescents and parents at post-intervention (T3). On a scale ranging from 1 to 10 (poor to excellent), adolescents and parents evaluated their subjective satisfaction with several elements of the training (i.e., whether the topics of the didactic lessons and/or the homework assignments covered their desires, the contact/alliance with the trainers, whether they would recommend the training to others, and their overall satisfaction).
- *2. Protocol Fidelity* Achieving treatment fidelity and consistency with the treatment manual is important in effectiveness studies. Adherence to treatment protocol was therefore monitored each session by trained research assistants during both the adolescent and parent PEERS® group sessions as well as the ROAD adolescent group sessions, through fidelity scoring sheets. Coverage of each part of the session material was scored on a 3 point-Likert scale (0%-not covered, 50%-partly covered, 100%-covered entirely). Overall fidelity was then operationalized as the total average percentage of the protocol that was covered by the clinicians in all sessions. Fidelity percentages were compared between the conditions to ensure that exposure to intervention was equal between conditions. In addition, members of the research team met regularly with the clinicians and coaches to review what had happened during the sessions, and to troubleshoot any clinical issues that may have arisen.
- *3. Compliance* In both intervention groups, trainers recorded adolescents and parent’s compliance with homework assignments and attendance, also expressed in an average percentage.
- *4. Group dynamics* It was noted if externalizing/aggressive behaviors caused severe disruptions during the group sessions. Problematic group dynamics were included as process variable if they occurred during at least two sessions in either condition. Group leaders were asked to indicate if a severe disturbance had occurred, and to report this to the study staff. Examples of severe disturbances may include physical encounters between group members, or verbally aggressive behaviours which resulted in exclusion of the participant that expressed the behaviour (in that session), and behaviour of group members resulting in an overall unsafe environment (as judged by the group leader).

### Statistical analyses

#### Attrition

Attrition analyses were performed firstly, in order to investigate if outcomes were not biased by potential selective attrition. We differentiate between study attrition (participants did not continue to take part in the assessments) and programme attrition, where participants stopped taking part in the intervention, but still took part in the assessments.

We used independent sample t-tests and Chi-square tests to compare baseline measures of age (adolescent and parent), sex (adolescent and parent), total IQ, performance and verbal IQ, as well as the total SRS-2 score, between: 1) those who had complete data on the CASS at baseline versus those who had missing data on the CASS at baseline, and 2) those who dropped out during the intervention versus those who completed the intervention (i.e. from pre to post). Then, 3) we examined whether there were significant differences between participants who only had pre- or post-assessment data versus participants who completed the assessments at all time-points (including follow-up). Finally, 4) we checked potential differences is treatment satisfaction between parents who only completed the SRS-2 at pre- and post-assessment versus parents who completed the SRS-2 at all-time points.

#### Descriptive statistics and comparison between conditions (i.e., covariate check)

Next, baseline characteristics (mean [SD]/frequency) of demographic, diagnostic fidelity/compliance and outcome measures were calculated for adolescents and parents in both conditions. Chi-square tests for dichotomous variables (i.e. (1) sex, 2) usage of psychiatric medication, and 3) previous social skills training) and independent *t*-tests for continuous variables (i.e. (1) adolescent and parent’s age, 2) ADOS-2 total calibrated severity score, 3) cognitive ability (IQ), and 4) outcome measures (i.e. CASS, CRS (both adolescent as well as confederate), SRS-2, SSIS) were conducted to determine whether there were significant differences between the PEERS® and ROAD groups. This was done to check whether variables needed to be added as covariates in the main analyses.

#### Main analyses

To compare the effects of the interventions (i.e., PEERS® versus ROAD) on the adolescents’ social skills in the primary outcome measure (CASS), we used Linear Mixed Model (LMM). LMM is advantageous compared to repeated measures ANOVA, because it accommodates missing time points, utilizing all available data, and therefore can be considered a true intention-to-treat model [35]. LMM uses maximum likelihood estimation to accommodate missing data. We used the data from 99 adolescents that had complete CASS at baseline. Time (three levels: baseline, post, and follow-up) was set as a repeated variable. Condition (two levels: experimental intervention and active control intervention) was added as a factor. We used an unstructured covariance matrix to allow unequal variances and covariances between random effects in the model. First, an unadjusted interaction between condition and time directly tested whether there was a significant difference between the two conditions over time. To correct for multiple testing, we used the false discovery rate (FDR) in the analyses on the subscales of the secondary outcome measures (SSIS and SRS-2) [36]. FDR is a way to correct for multiple testing, in which the proportion of false discoveries is calculated as a reference to all significant results. It hereby limits the number of false positives reported as significant. It is considered a powerful alternative to the traditional approach of avoiding even a single false discovery. FDR has been advocated to increase the ecological validity of studies [37]. The correction followed a stepwise procedure, whereby all *p*-values of a test were sorted in ascending order to create a ranking (small – large). The total number of *p*-values was divided by the rank of a specific *p*-value and then multiplied by the original *p*-value.

#### Process information

Mean overall treatment satisfaction was calculated for parents and adolescents in both conditions. To compare the adolescent and parent’s satisfaction and homework compliance between the two conditions, we performed independent *t*-tests.

#### Post hoc* analyses*

In case of a significant group-by-time effect of the intervention(s) on the primary or secondary outcome measures, we explored whether these could be explained by treatment satisfaction. More specifically, we explored whether the difference scores (i.e., from pre to post and from post to follow-up) of the SRS-2 parent and SSIS adolescent were correlated with treatment satisfaction.

All statistical analyses were performed using SPSS version 25 (IBM, Corp, Armonk, NY, USA).

## Results

### Participants and assessments

**Fig. 1:**
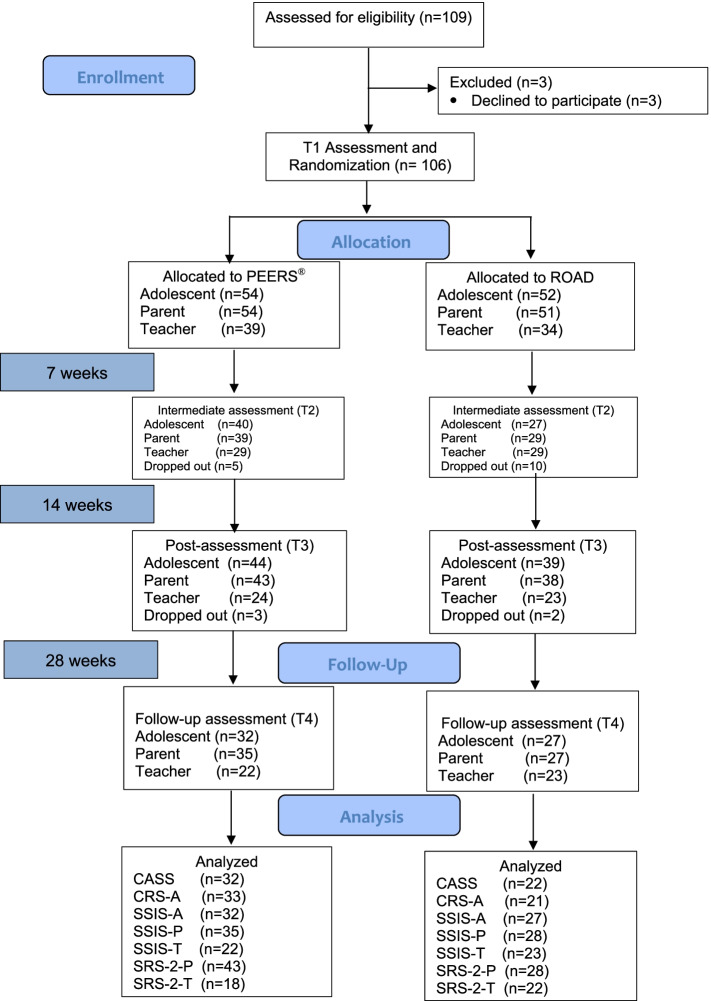
CONSORT Flow Diagram

Figure 1 provides the CONSORT Flow Diagram of the current study, illustrating participants’ movement throughout the study. A total of 109 adolescents engaged in the current study. During baseline assessment, three adolescents withdrew their further participation and were thus excluded. In the randomization procedure, the remaining 106 participants were randomly assigned to the experimental treatment condition group (PEERS® *n* = 54) or the active control condition group (ROAD *n* = 52). Of these 106 participants, 83 (78%; *n* = 44 PEERS®, *n* = 39 ROAD) participated at the post-assessment (T3), and 59 (56%; *n* = 32 PEERS®, *n* = 27 ROAD) participated at the follow-up (T4). Parents participated concurrently in addition to their child; 105 parents (*n* = 54 PEERS®, *n* = 51 ROAD) completed baseline assessment (T1), of which 81 parents (76%; *n* = 43 PEERS®, *n* = 38 ROAD) participated at the post-assessment (T3), and 62 parents (58%; *n* = 35 PEERS®, *n* = 27 ROAD) participated at the follow-up (T4).

In total, 19% of the 106 participants (in total *n* = 20; of which *n* = 8 in the experimental treatment condition and *n* = 12 active control condition) dropped out of the interventions due to a lack of motivation (*n* = 6), the intervention not fitting the adolescents’ and/or parents’ desires (*n* = 6), not being comfortable with the group format (*n* = 3), being overstrained with school requirements (*n* = 2), family problems (*n* = 2), or logistic time/travel problems (*n* = 1).

### Attrition

All attrition analyses outcomes are described in additional file 2. The only significant finding indicated that younger participants were more inclined to have missing CASS data at T1. Further analyses did not reveal any selective attrition in the study or the interventions.

### Descriptive statistics and comparison between conditions

The baseline characteristics of the adolescents are presented in Table 1. A total of 73 males and 33 females participated (Note, this concerns biological sex; two participants were trans-gender, i.e., transitioning from female to male sex throughout the duration of the study), with a mean age of 14.56 (SD = 1.55) years old. Participants had a mean total IQ of 103.44 (SD = 13.95), with a range of 79 to 135 and a mean ADOS calibrated severity score of 5.55 (SD = 2.45), ranging from 1 to 10. 43% of parents reported that their child was currently using psychotropic medication, such as an SSRI or methylphenidate, and 60% of the adolescents previously had a social skills training (e.g., during childhood).

**Table 1:** RCT group comparison on demographics and baseline assessment measures

	PEERS® (*n* = 54) M (SD)	ROAD (*n* = 52) M (SD)	*t or X*^*2*^	*p*
**Demographic & Diagnostic info**				
Adolescent age (years)	14.65 (1.51)	14.48 (1.62)	.55	.58
Adolescent sex (m:f)	39:15	34:18	.58	.45
Parent age (years)	47.81 (6.90)	47.15 (6.24)	.52	.61
Parent sex (m:f)	12:42	11:41	.02	.89
SRS-2 score Parents	57.46 (12.40)	56.22 (12.05)	.50	.62
Usage of psychiatric medication (yes:no)	22:31	23:29	.08	.78
Previous social skills training (yes:no)	34:20	30:21	1.24	54
WISC/ WASI Total	101.47 (20.68)	103.98 (12.40)	-.67	.50
WISC/ WASI Performance	103.28 (14.62)	107.07 (10.65)	.21	.83
WISC/ WASI Verbal IQ	100.88 (16.84)	100.15 (14.99)	-1.35	.18
ADOS-2 CSS	5.31 (2.54)	5.77 (2.39)	-.81	.42
**Process information**				
Compliance Attendance	A:89.35% P:87.01%	A: 83.28%	-1.90	.06
Compliance Homework	A:74.86% P:70.78%	A: 68.55%	-1.60	.11
Fidelity	A: 92.87% (6.17) P: 97.52% (2.81)	A: 92.63% (6.72)	-.08	.90
Group dynamics – Problematic disturbances, % of participants involved (i.e. externalizing/aggressive behaviors) during more than 2 group sessions	7.3%	12.8%	.14	.16
Treatment Satisfaction (Parent)	8.20 (1.46)	7.52 (1.45)	1.97	.05*
Treatment Satisfaction (Adolescent)	7.51 (1.67)	7.28 (2.46)	.51	.61
	(*n* = 48) M (SD)	(*n* = 41) M (SD)		
CASS total confederates	37.41 (5.01)	36.98 (4.27)	.44	.66
CRS total confederates	26.23 (5.77)	23.67 (5.71)	2.23	.03*
**T1 Adolescent measures**				
SSIS-A total	84.31 (20.45)	85.51 (16.43)	-.33	.74
CASS total	30.13 (8.22)	28.91 (7.10)	.75	.46
CRS-A total	27.45 (4.46)	25.60 (4.77)	2.01	.04*
**T1 Parent measures**				
SRS-2-P total	88.90 (27.09)	86.20 (22.02)	.56	.58
SSIS-P total	70.26 (18.49)	73.20 (16.35)	-.86	.39
**T1 Teacher measures**	*n* = 37	*n* = 31		
SRS-2-T total	77.92 (24.51)	73.94 (26.71)	.64	.52
SSIS-T total	69.90 (17.98)	71.59 (15.76)	-.42	.67

*p* < .05 *, *p* < .01 **

Contextual Assessment of Social Skills (*CASS*); Social Skills Improvement System (*SSIS*); Social Responsiveness Scale-2 (*SRS-2*); Conversation Rating Scale (*CRS*); Wechsler Intelligence Scale for Children (*WISC*); Wechsler Abbreviated Scale of Intelligence (*WASI*); Autism Diagnostic Observation Schedule-2 (*ADOS-2*)

There were no significant differences in the demographic, diagnostic, fidelity/compliance, or the baseline assessments of all outcome measures between the PEERS® and the ROAD condition. In the PEERS® condition, in one group severe disturbances occurred (i.e., externalizing, aggressive behaviors) during more than 2 sessions, while in the ROAD condition this was the case in 2 groups. This reached however no significant difference between the two conditions.

The self-reported score of the total CRS differed significantly between conditions. More specifically, at baseline, adolescents in the PEERS® condition perceived more interest in the conversation by the confederate (M = 27.45, SD = 4.46) than the adolescents in the ROAD condition (M = 25.60, SD = 4.77). Also, CRS scores were significantly associated with a few SRS and SSIS variables across groups. Therefore, the baseline CRS score was added as a covariate in all main analyses. A significant difference was also found in the CRS scores of the confederates at baseline. However, since CRS scores of the participants and those of the confederates were significantly correlated (*r* = 0.27, *p* = 0.007), we only controlled for the CRS scores of the participants in the further LMM.

### Effectiveness of the interventions

- 1. *Primary outcome:* No significant condition by time interactions were found for the CASS and CRS variables (see Table 2).
  As shown in Additional file 3, significant main effects of time were found for positive affect (*p* = 0.03), overall quality of rapport (*p* = 0.05), and ending conversation (*p* < 0.01). Starting a conversation had a significant time effect and group effect, both groups also improved over time, but the interaction was not significant. CRS Total and Perspective taking questions showed a time effect, also irrespective of condition (*p* < 0.01). The scores in both groups increased over time, which means that over time, the adolescents perceived more interest from their conversational partner in the conversation and believed that the conversation was more pleasant for their conversational partner.
- *2. Secondary measures of social skills*

**Table 2:** Descriptives and main results Linear Mixed Model analyses (*N* = 106)

	**PEERS**®			**ROAD**			**LMM analysis interaction *CONDITION X TIME***
	Baseline M(SD)	Post M(SD)	Follow-up M(SD)	Baseline M(SD)	Post M(SD)	Follow-up M(SD)	*p*
**Adolescent data**							
	*n* = 53	*n* = 42	*n* = 32	*n* = 46	*n* = 35	*n* = 22	
**CASS Total**	30.13 (8.22)	30.21 (8.29)	33.63 (9.15)	28.91 (7.99)	30.46 (7.98)	29.59 (7.31)	.18
0. Starting conversation	1.62 (0.71)	1.86 (0.47)	1.66 (0.60)	1.35 (0.82)	1.74 (0.61)	1.68 (0.65)	.18
1. Total Questions Asked	6.19 (3.54)	7.02 (4.24)	6.50 (3.29)	6.24 (4.14)	6.51 (3.50)	5.64 (3.26)	.70
1a. Initiating Questions	2.92 (1.89)	3.50 (1.64)	3.03 (1.68)	2.78 (1.95)	3.66 (1.94)	3.09 (1.88)	.87
1b. Follow-up Questions	3.26 (2.68)	3.52 (3.29)	3.47 (2.76)	3.46 (3.35)	2.89 (2.70)	2.45 (2.26)	.45
2. Topic Changes	3.06 (2.22)	2.93 (1.80)	3.47 (1.90)	3.24 (2.38)	3.40 (2.55)	3.27 (2.12)	.56
3. Vocal Expressiveness	4.91 (1.56)	5.12 (1.49)	5.22 (1.66)	4.67 (1.61)	4.80 (1.61)	4.32 (1.62)	.21
4. Gestures	3.47 (1.86)	3.14 (2.06)	4.09 (1.91)	3.24 (1.88)	3.17 (1.92)	3.23 (1.95)	.26
5. Positive Affect	4.32 (1.54)	4.57 (1.63)	5.16 (1.76)	4.07 (1.53)	4.51 (1.54)	4.45 (1.41)	.42
6. Kinetic Arousal	4.02 (1.66)	3.95 (1.55)	4.09 (1.75)	3.98 (1.15)	3.97 (1.40)	3.95 (1.17)	.62
7. Social Anxiety	4.53 (1.68)	4.33 (1.79)	4.81 (1.62)	4.09 (1.74)	4.77 (1.54)	4.36 (1.36)	.48
8. Overall Involvement	4.62 (1.30)	4.71 (1.45)	5.22 (1.26)	4.70 (1.31)	4.94 (1.24)	4.82 (1.05)	.29
9. Overall Quality of Rapport	4.26 (1.48)	4.38 (1.43)	5.03 (1.49)	4.17 (1.39)	4.29 (1.47)	4.45 (1.44)	.27
10. Initiating End of Conversation	0.60 (0.49)	0.95 (0.22)	0.84 (0.37)	0.67 (0.60)	0.94 (0.24)	0.95 (0.21)	.75
11. Giving reason to end conversation	0.26 (0.45)	0.45 (0.50)	0.41 (0.50)	0.24 (0.43)	0.37 (0.49)	0.32 (0.48)	.71
	*n* = 53	*n* = 42	*n* = 33	*n* = 47	*n* = 36	*n* = 21	
**CRS-A**	27.45 (4.46)	27.67 (5.38)	28.91 (3.25)	25.60 (4.77)	27.61 (4.80)	28.52 (3.63)	.12
Item 1 (self-confidence before)	4.66 (1.41)	4.88 (1.61)	5.09 (1.56)	4.52 (1.87)	4.62 (1.79)	4.84 (1.95)	.07
Item 10 (after)	4.85 (1.42)	5.18 (1.37)	5.23 (1.31)	4.43 (1.92)	4.82 (1.74)	5.11 (1.79)	.42
Perspective taking items	10.16 (1.76)	10.64 (2.25	11.26 (1.44)	9.96 (1.91)	10.42 (1.84)	10.53 (2.29)	.50
	*n* = 52	*n* = 45	*n* = 32	*n* = 52	*n* = 39	*n* = 27	
**SSIS-A Total**	82.33 (21.91)	90.82 (20.26)	94.53 (23.02)	84.96 (16.33)	88.85 (18.12)	88.11 (21.43)	.02*
Communication	12.00 (3.30)	13.77 (2.94)	13.53 (3.59)	12.67 (2.78)	12.97 (2.64)	13.54 (3.14)	.06
Cooperation	14.59 (3.45)	15.42 (3.54)	16.43 (3.45)	15.14 (3.10)	15.08 (3.35)	14.60 (3.67)	.01#
Assertion	12.06 (4.03)	12.16 (3.73)	12.69 (4.34)	11.10 (4.17)	12.11 (3.92)	12.41 (4.45)	.38
Responsibility	13.42 (3.38)	14.66 (3.18)	15.30 (3.49)	13.46 (2.79)	13.56 (3.30)	14.50 (3.28)	.33
Empathy	12.29 (3.39)	12.95 (3.41)	13.48 (3.29)	12.84 (3.10)	13.36 (2.59)	12.35 (3.19)	.12
Engagement	9.46 (3.92)	11.09 (4.59)	11.90 (4.91)	9.25 (4.62)	10.68 (4.59)	10.42 (5.14)	.02#
Self-Control	10.65 (4.16)	12.26 (2.80)	12.65 (3.21)	10.79 (3.66)	11.51 (4.26)	11.80 (4.25)	.53
**Parent data**							
	*n* = 54	*n* = 41	*n* = 35	*n* = 51	*n* = 38	*n* = 28	
**SSIS-P Total**	70.26 (18.49)	80.90 (17.47)	84.83 (21.79)	73.20 (16.35)	83.39 (19.91)	82.93 (18.02)	.11
Communication	9.79 (3.32)	11.56 (3.17)	12.29 (3.41)	10.40 (2.60)	11.42 (2.94)	11.48 (2.58)	< .01**
Cooperation	10.08 (3.50)	11.63 (3.17)	11.91 (3.97)	10.92 (3.03)	12.38 (3.24)	12.50 (3.09)	.28
Assertion	10.57 (3.38)	11.49 (3.27)	11.24 (3.55)	10.63 (3.19)	11.55 (3.21)	11.54 (3.10)	.97
Responsibility	9.98 (3.02)	11.61 (3.27)	11.97 (3.69)	10.28 (3.23)	11.92 (3.74)	12.64 (3.07)	.89
Empathy	9.94 (2.95)	10.85 (2.56)	11.91 (3.27)	10.25 (3.21)	11.55 (2.78)	11.25 (3.25)	.11
Engagement	8.04 (3.96)	9.88 (3.78)	10.37 (4.36)	8.39 (3.51)	10.61 (4.20)	10.43 (4.11)	.40
Self-Control	7.31 (3.40)	8.46 (2.99)	8.77 (3.64)	8.08 (3.11)	8.79 (3.57)	8.78 (3.51)	.41
	*n* = 54	*n* = 51	*n* = 43	*n* = 39	*n* = 35	*n* = 28	
**SRS-2-P Total**	88.90 (27.09)	69.09 (27.15)	62.26 (25.06)	86.20 (22.02)	72.76 (26.18)	69.95 (23.72)	.02*
Social Awareness	11.44 (3.13)	9.95 (3.66)	8.91 (3.39)	11.18 (3.13)	9.32 (3.68)	9.07 (3.30)	.58
Social Cognition	16.67 (6.59)	13.30 (6.49)	12.29 (6.13)	15.22 (5.34)	13.53 (5.08)	12.64 (5.65)	.03^#^
Social Communication	29.17 (10.11)	22.02 (9.99)	19.91 (9.98)	27.75 (8.94)	22.89 (10.62)	22.89 (9.77)	.04^#^
Social Motivation	16.56 (6.27)	11.91 (6.51)	10.63 (5.51)	16.75 (4.46)	13.08 (5.51)	12.36 (4.52)	.15
Autistic Mannerisms	14.96 (6.35)	11.65 (5.62)	10.37 (5.05)	14.78 (5.53)	12.18 (5.39)	12.14 (6.13)	.05^#^
**Teacher data**							
	*n* = 39	*n* = 24	*n* = 22	*n* = 34	*n* = 23	*n* = 23	
**SSIS-T Total**	69.90 (17.98)	66.38 (17.88)	73.36 (18.85)	71.59 (15.76)	78.26 (17.99)	75.39 (15.74)	.31
Communication	11.85 (3.89)	11.83 (3.62)	12.36 (4.26)	12.15 (3.63)	13.39 (3.69)	12.17 (3.20)	.45
Cooperation	11.79 (2.82)	11.17 (2.57)	12.41 (2.75)	12.91 (3.33)	12.78 (3.67)	12.30 (3.11)	.07
Assertion	9.13 (3.70)	8.63 (3.92)	9.05 (4.25)	8.09 (3.25)	9.09 (2.54)	9.22 (3.50)	.49
Responsibility	11.67 (2.97)	10.38 (2.18)	11.77 (2.33)	12.21 (3.68)	12.96 (4.17)	12.22 (3.20)	.27
Empathy	7.90 (3.70)	6.92 (3.67)	8.55 (3.42)	7.18 (3.66)	8.65 (3.70)	8.57 (2.35)	.71
Engagement	7.74 (3.70)	8.08 (3.43)	8.77 (4.13)	8.15 (3.59)	9.78 (3.90)	8.48 (4.40)	.16
Self-Control	9.82 (3.87)	9.39 (3.16)	10.45 (3.08)	10.91 (4.20)	11.61 (3.37)	12.43 (3.44)	.33
	*n* = 37	*n* = 22	*n* = 18	*n* = 31	*n* = 20	*n* = 22	
**SRS-2-T Total**	77.92 (24.51)	72.64 (24.01)	65.11 (30.89)	73.94 (26.71)	63.30 (21.27)	65.09 (20.66)	.58
Social Awareness	9.68 (3.70)	9.09 (2.30)	8.67 (2.97)	9.35 (3.47)	8.30 (3.31)	8.59 (3.33)	.45
Social Cognition	15.05 (4.84)	15.50 (4.98)	14.67 (5.35)	15.13 (5.38)	12.50 (3.75)	13.09 (3.21)	.66
Social Communication	26.95 (10.01)	25.05 (9.90)	21.39 (12.37)	25.71 (10.71)	22.25 (9.32)	22.73 (7.74)	.27
Social Motivation	14.76 (4.90)	13.23 (5.61)	12.39 (6.44)	13.74 (5.42)	12.00 (4.44)	12.00 (5.22)	.81
Autistic Mannerisms	11.49 (5.44)	9.77 (5.13)	8.00 (6.12)	10.00 (5.81)	8.25 (4.94)	8.68 (5.04)	.54

^*^p < .05, no FDR correction applied, # not significant after FDR correction, ** Remained significant after FDR correction

CRS as covariate in analyses on all measures

Contextual Assessment of Social Skills (*CASS*); Social Skills Improvement System (*SSIS*); Social Responsiveness Scale-2 (SRS-2); Conversation Rating Scale (*CRS*)

Adolescent self-reports (SSIS-A Total) in the PEERS® group showed a significantly greater increase in overall social skills as compared to the ROAD group, F(1, 137.86) = 5.37, *p* = 0.02. After FDR correction, the effects on the subscales cooperation F(1, 142.47) = 6.99, *p* = 0.01, and engagement F(1, 130.77) = 5.23, *p* = 0.02 did not remain significant. Additionally, parent-reported communication of their child (SSIS subscale) increased significantly more in the PEERS® group than in the ROAD group F(1, 138.79) = 7.20, *p* < 0.01. A significant main effect of time was found on the SSIS Total as reported by parents F(1, 142.17) = 29.26, *p* < 0.01, indicating that adolescents in both groups significantly improved their parent-reported social skills over time, regardless of condition (Additional file 2).

A significant treatment effect was found on the total score of social skill impairment (SRS-2), F(1, 141.72) = 5.63, *p* = 0.02, meaning that parental reports on the social skills impairment of the adolescents in the PEERS® group decreased significantly more than in the ROAD condition. This effect was driven by the specific subscales of social cognition F(1, 137.58) = 5.01, *p* = 0.03, social communication F(1, 145.97) = 4.28, *p* = 0.04, and autistic mannerism F(1, 142.80) = 3.80, *p* = 0.05, of which the specific effects however did not remain significant after the FDR correction. Meanwhile, teacher-reported total SRS-2 of participants in both groups significantly decreased over time, regardless of condition F(1, 58.52) = 6.79, *p* = 0.01 (please see Table 2 for means and Additional file 3 for time effects).

### Treatment satisfaction

On average, adolescents and their parents in both groups reported high overall satisfaction with the interventions: Parents in the PEERS® condition (M = 8.20, SD = 1.46) reported significantly higher satisfaction than parents in the ROAD condition (M = 7.52, SD = 1.45). Teens did not differ significantly with regard to satisfaction; PEERS® (M = 7.51, SD = 1.67) versus ROAD (M = 7.28, SD = 2.46). Amongst the adolescents who completed the interventions, 89.35% of the PEERS® participants versus 83.28% of the ROAD participants attended at least 12 out of 14 training sessions. The overall homework completion rate was 74% in the PEERS® condition versus 68% in the ROAD condition. No significant differences were found between conditions with regard to compliance and fidelity.

## Discussion

In the current study, we used a randomized controlled trial design with an experimental intervention condition versus an active control condition, to examine the effectiveness of the Dutch culturally adapted version of PEERS®, in improving social skills amongst adolescents with ASD. Direct intervention outcomes and maintenance were investigated using behavioral observation as the primary outcome measure of social skills. In addition, self-, parent-, and teacher reported questionnaires were used as indices of social skills. Moreover, participants satisfaction was assessed.

Results of the primary outcome concerning the behavioral observation (CASS) did not reveal significantly greater improvements in the adolescents who received the PEERS® intervention, as compared to those who received the ROAD intervention. However, regardless of the intervention condition, significant improvements over time were found on ‘positive affect’, the ‘overall quality of rapport’, ‘starting a conversation’, and ‘taking initiative to end the conversation’ domains, suggesting improvements in the adolescents’ abilities in these domains over time. In these analyses we controlled for the CRS scores of participants at baseline, to ensure that differences between conditions in the subjective experiences of participants at baseline did not influence the main result.

This was the first study to use an active control condition rather than a waiting list control condition to examine the effectiveness of PEERS®. As such, we replicated and extended earlier research using the CASS [6, 7, 11, 12, 38]. Dolan et al. [13] also reported a trend for improvement on quality of rapport from pre- to post PEERS® intervention. Moreover, Rabin et al. [38] found a significant change following the intervention on the CASS in the immediate and the delayed condition. Arguably, in our study, behaviors to establish rapport were developed during both interventions, indicating existence of non-specific treatment effects such as belongingness to a like-minded therapeutic group, or different working mechanisms leading to similar outcomes. Note that social skills (verbal and non-verbal behaviors) were explicitly practiced in various contexts and homework assignments throughout the duration of PEERS®, but not in ROAD. Although ROAD does not directly or explicitly train verbal and non-verbal skills like building rapport, it does direct self-knowledge, self-acceptance, and the sense of belongingness to a specific ‘neurotribe’, which probably makes adolescents more comfortable with who they are, sure to remain authentic and self-confident. Self-confidence is expressed non-verbally through positive affect (eye-contact, smiling, open posture) and can make the conversation more relaxed, improving rapport. Self-confidence can also help in more pro-actively starting and finishing a conversation. Thus, although the CASS formally assesses social skills, the observed behaviors can also be considered indices of self-confidence, one of the treatment goals of ROAD. Thus, although both interventions each target their own mechanisms and outcomes (PEERS®: social skills, ROAD: self-knowledge and self-confidence), they seem to result in similar observable outcome behaviors. With regard to this particular outcome, PEERS® is not superior to ROAD. That being said, secondary measures of social skills did indicate superiority of PEERS® in the domain of social skills.

With regard to these secondary measures, we used questionnaire data from multiple informants, as indices of the construct of social skills. We found more pronounced improvements in self-reported social skills (i.e., SSIS total scale) in the PEERS® intervention as compared to the ROAD intervention. More specifically, this effect was mainly driven by the cooperation and engagement subscales, although these did not remain significant if regarded in isolation using FDR corrections. PEERS® stimulates joint activities [39], therefore effects on these traits were to be expected. It is of added value that adolescents themselves also perceived these improvements.

According to the parents’ SSIS reports, PEERS® had an immediate and longer-term effect on social skills (e.g., improvement). Our RCT findings show a significant time effect on the SSIS parent, in both intervention groups. The group-by-time interaction effect on the SSIS parent subscale showed that specifically the communication of adolescents in PEERS® group improved and maintained until at least 14-weeks after completing the intervention, which was not the case in the ROAD group. The PEERS® intervention aims to develop adolescents’ verbal and nonverbal reciprocal communication skills. During the intervention, they learn and practise how to trade information, how to participate in a two-way reciprocal conversation, and how to identify and to use nonverbal cues. These skills were built up and enhanced communication abilities that were maintained at follow-up.

Also, adolescents in the PEERS® group showed a significant reduction in the parent reported SRS-2 total score, as compared to adolescents in the ROAD group, which was driven by the subscales social communication, social cognition and autistic mannerisms. These results were comparable to those in most previous studies [7–10, 12, 38]. The significant reductions were maintained at the 14-week follow-up. This maintenance effects might be attributed to the enhanced parental skills and parental involvement as a social coach to their adolescents, which continued after completing the training.

Also according to teachers, the SRS-2 scores significantly reduced over time, which however did not differ significantly between the conditions. We suspect that the low response rate of teachers (i.e., reducing statistical power) might have affected the results. The low response rates of teachers have also been reported in similar studies [7, 8, 10–12, 38]. Despite repeated efforts to remind teacher to complete the questionnaires online, response rates were still far from satisfactory. Some of the teachers that participated in the study reported about their poor familiarity with specific social behaviors that were mentioned in the questionnaires. Other reasons were that the adolescent had finished school or was not in their class anymore, or time constraints due to the burden of workload.

Finally, parents in the PEERS® group were more satisfied with the intervention compared to parents in the ROAD group. Meanwhile, adolescents in both groups did not differ in their evaluation of the interventions. In addition, satisfaction scores were not associated with changes in outcome in SRS-2 and SSIS, suggesting that improved social skills did not result mainly out of satisfaction of parents, but probably reflected actual progression.

### Methodological and clinical considerations

This study added to the cross-cultural validation of PEERS*®*. The strength of our methodological approach is four-fold. First, this is the first study to compare PEERS® to an active treatment control condition, instead of to a waiting list control condition (as previously been used in [6–9, 12, 13]). An active treatment control condition allows for the identification of treatment effects that are specific to PEERS®. Second, we added self-reported data on social skills. Third, an objective observation, the CASS [18], was used as our primary outcome measure. Finally, participant’s and parents satisfaction were assessed. 

A few limitations should however, also be mentioned. First, in our study we used the behavioral observation measure, CASS, in order to objectively observe the effectiveness of the intervention on social behavior. However, the logistics and implementation of the CASS are rather complex and time-consuming. For instance, it is hard to obtain suitable confederates and to train coders to score the CASS reliably. Ratto et al. [18] introduced the CASS as a laboratory-based observational measure of social skills. As such, it currently does not meet the feasibility requirements for use in daily clinical practice in determining social skills or improvement in social skills following intervention. Additionally, the CASS can be quite stressful for the participants, making the instrument unnaturally charged, whereby the outcome of the instrument is not merely an outcome of social skills, but also of the self-management of (social) stress/arousal. We did however control for such subjectively experienced stress levels in our RCT by controlling for the CRS scores of participants in our analyses, which did not alter our findings with the CASS or secondary outcomes.

Second, our study suffered from dropouts. In total, 19% of the participants dropped-out from the interventions. However, attrition analysis mostly did not reveal selective attrition, except for CASS completers being slightly older. Previous studies reported slightly lower to similar drop-out rates, e.g., 14.6% [39], 13% [38], and 18% [8], yet these studies we not conducted in a specialized mental health care setting (i.e., higher comorbidity rates) like the current study.

Third, the PEERS® adolescent intervention focusses on motivated adolescents without intellectual disability. This limits the generalizability of our findings to the broader population of adolescents with ASD (i.e., with limited motivation and/or limited cognitive abilities).

### Conclusion, summary, and directions for future research

We found suggestive evidence that the Dutch cultural adaptation of the PEERS® parent-assisted intervention for adolescents with ASD is an effective program to improve social skills. With these findings, we extend the cross-cultural validation of this program. Implementation in the Dutch mental care system will be a next step to further tackle the social challenges of adolescents with ASD.

Our findings also partly replicate previous research, where a positive effect of the PEERS® training was also observed as improvement in social skills of adolescents (as measured by parent and self-reports on the SRS-2 and CASS). Our study adds to these previous studies, by comparing to an active treatment control condition, with some social skill outcomes improving more in the PEERS® intervention compared to an active control condition. The results suggest the existence of other (yet unknown) active or nonspecific mechanisms that influence the effectiveness of the intervention.

ROAD also seems a valuable psycho-education program, potentially boosting self-confidence. More research is however needed to ascertain the working mechanisms and effectiveness of ROAD.

Our findings suggest that the PEERS® intervention does have a superior effect on parent and self-reported social skills compared to the active treatment control condition. Also, treatment satisfaction of parents was higher in the PEERS® group, suggesting that the PEERS® treatment was a good fit with the needs of parents who are raising an adolescent with ASD.

Despite the limitations in our study, the results provide indications that PEERS® as a parent-assisted social skills training program is efficacious in enhancing the social communication skills of Dutch adolescents with ASD. Yet, further research is needed as to how these effects can be optimized. In future studies, not only the sheer effectiveness of interventions should be investigated, but it should be further explored for whom the PEERS® or ROAD intervention work best (moderators), and through which causal chains of change (mediators).

## Supplementary Information

**Additional file 1.** Overview of RCTs examining the effectiveness of PEERS® on adolescent behavioral outcomes.

**Additional file 2.** Attrition analyses outcomes.

**Additional file 3.** Results Linear Mixed Model analyses; main effects of time and condition (*N* = 106).

## Data Availability

The datasets generated and/or analyzed during the current study are not publicly available but, are available from the corresponding author on reasonable request after the results are published. The policy of Erasmus Medical Centre is that sharing data with parties outside the organization requires a personalized data transfer agreement between Erasmus Medical Centre and these parties. This requirement prohibits publishing data in freely accessible depositories. Only authors have access to the data.

## Abbreviations

ADOS: Autism Developmental and Observation Schedule
ASD: Autism Spectrum Disorder
CASS: Contextual Assessment of Social Skills
CRS: Conversation Rating Scale
DSM: Diagnostic and Statistical Manual of Mental Disorders
LMM: Linear Mixed Model
PEERS®: Program for the Education and Enrichment of Relational Skills
RCT: Randomized Controlled Trail
ROAD: Regulation, Organization and Autonomy Didactics
SRS -2: Social Responsiveness Scale – version 2
SSIS: Social Skills Improvement System
WASI: Wechsler Abbreviated Scale of Intelligence
