# Supplementary material for: A randomized controlled trial to examine the effectiveness of the Dutch version of the Program for the Education and Enrichment of Relational Skills (PEERS®)
Source: BMC Psychiatry. 2022 Apr 22;22:293. doi: 10.1186/s12888-022-03913-3 (PMC9034592; doi:10.1186/s12888-022-03913-3)
Supplement: Supplementary file 1 — Additional file 1. Overview of RCTs examining the effectiveness of PEERS® on adolescent behavioral outcomes. [file 12888_2022_3913_MOESM1_ESM.docx]

*Additional file 1* Overview of RCTs examining the effectiveness of PEERS^®^ on adolescent behavioral outcomes

| Author | Design | N (EXP/CON) | In/exclusion criteria | Follow-up | Outcome measures | Results | Limitations |
| --- | --- | --- | --- | --- | --- | --- | --- |
| Laugeson et al., 2009^a^ | waitlist control group | EXP: n = 17  CON: n = 16 | - Age 13-17 years - Social problems - ASD diagnosis - English fluency - Verbal IQ > 70 - No history of major mental illness^b^ - No hearing, visual or physical impairments | no | Adolescent:   - **TASSK** - **QPQ host** - QPQ invite - QPQ conflict - **FQS**   Parent:   - QPQ host - QPQ invite - QPQ conflict - SSRS problem behaviors - **SSRS social skills**   Teacher:   - SSRS problem behaviors - SSRS social skills | - ***p* < .001** - ***p* < .025** - ns - is - ***a* < .05** - ns - ns - ns - ns - ***p* < .05** - ns - ns | - 12 weeks program - No follow-up - No teacher reports - Limited diagnostic assessment and burden |
| Laugeson et al., 2012 | waitlist control group | EXP: n = 14  CON: n = 14  groups of 8-10 | - Age 12-17 years - Social problems - ASD diagnosis - English fluency - Verbal IQ > 70 - No history of major mental illness^b^ - No hearing, visual or physical impairments | 14-week | Adolescent:   - **TASSK** - **QPQ host**   Parent:   - **QPQ host** - SSRS problem behaviors - **SSRS social skills** - **SRS total**   Teacher:   - SRS - SSRS social skills - SSRS assertion | - ***p* < .001** - ***p* < .015** - ***p* < .001** - ns (at follow-up *p* <.04) - ***p* < .001** - **<.02** - ns - ns (at follow-up <.03) - ns (at follow-up <.02) | - Lack of comprehensive diagnostic - Poor response rate from teacher - Long term follow-up |
| Schohl et al., 2014 | waitlist control group | EXP: n = 29  CON: n = 29  per group <10 | - Age 11- 16 years - Social problems - English fluency - No history of major mental illness^b^ - No hearing, visual or physical impairments - ASD diagnosis with ADOS - Verbal IQ > 70 | no | Adolescent:   - **TASSK** - **QSQ host** - **QSQ invite** - QSQ conflict - FQS - **SIAS**   Parent:   - QSQ host - QSQ invite - QSQ conflict - **SRS** - **SSRS problem behaviors** - SSRS social skills   Teacher:   - SRS - **SSRS problem behaviors** - SSRS social skills | - ***p* = .001** - ***p* = .005** - ***p* = .01** - ns - ns - ***p* = .01** - ns - ns - ns - ***p* = .005** - ***p* = .05** - ns - ns - ***p* = .05** - ns | - Lack of diversity - Parent rating leads to bias - Poor response rate from teacher |
| Yoo et al., 2014 | waitlist control group | EXP: n = 23  CON: n = 24  groups of 6-10 | - Age 12-18 years - Between sixth grade of elementary school to third grade of high school - Social problems - Motivated - No history of major mental illness^b^ - No aggressive behavior or severe oppositional tendency - No hearing, visual or physical impairments - No other neurological /physical illnesses - ASD diagnosis - Verbal IQ ≥ 65 | 3 months | Adolescent:   - **TASSK-R** - QPQ host - QPQ invite - QPQ conflict - **CDI** - STAIC-T - STAIC-S   Parent:   - **QPQ host** - QPQ invite - QPQ conflict - **VABS** - SRS - SCQ current - ASDS - SSRS problem behaviors - SSRS social skills - **CBCL**   Clinician:   - **ADOS** | - **p < .01** - ns (if controlled   p =.04)   - ns - **p = .04** - ns - ns - **p =.06** - ns - ns - **p <.01** - ns - ns - ns - ns - ns - **p = .02 (internalizing)** - **p =.01** | - No direct observation data - ADOS was partially blinded, administrator should be blinded to condition - No teacher rating - Treatment acceptability measures |
| Dolan et al., 2016 | waitlist control group | EXP: n = 28  CON: n = 30 | see Schohl et al. 2013 | No | Adolescent:   - **TASSK**   Observation:   - **CASS** | ***p* < .05**; *η*_p_^2^ = .77  only vocal expressiveness *p* < .05 | - Small and limited samples - Laboratory setting elicit less natural responses - Not a natural instruction (playing Jenga or plying puzzle) - Confederate behave only interested condition |
| Rabin et al., 2018^c^ | Waitlist  Control group | EXP: n = 20  CON: n = 21 | - Ages 12-17 years - ASD diagnosis - Total IQ > 70 - No severe behavioral problems - Motivated - One parent willing to serve as social coach | 16-week | Adolescent:   - **TASSK** - **QSQ host** - **QSQ invite** - LSDQ - EQ - **CASS**   Parent:   - QSQ - **SSIS social skills** - **SSIS behavioral problems** - **SRS-2**   Teacher:   - SSIS - SRS | - ***p* <.001** - ***p* < .01** - ***p* < .05** - ns - ns - CASS total , asking questions *p* < .05, overall involvement *p* < .05 at follow-up - ns - ***p* < .01** - ***p* < .05** - ***p* < .01** - ns - ns | - Samples included only 2 girls - Parents and teachers were not blinded to randomization - Small samples |
| Shum et al., 2018 | Waitlist  Control group | EXP: n = 38  CON: n = 34 | - Ages 11-15 years - Fluent in Cantonese - Secondary school - Social difficulties - ASD diagnosis - VIQ > 70 - Moderate to severe deficits in social interactions based on ADOS - Motivated - No hearing, visual or physical impairments - No history of major mental illness^b^ | 14-week | Adolescent:   - **TASSK** - QPQ-A   Parent:   - ABAS communication - ABAS social - **ABAS self-direction** - **SRS** - QPQ-P   Teacher:   - SRS   Peer:   - ABAS | - ***p* < .001** - ns - ns - ns - ***p* < .05** - ***p* < .01** - ns - ns - ns | - No treatment integrity data - Poor response rate from teacher and peers - No direct observation |
| Yamada et al., 2019 | Waitlist  Control group | EXP: n = 14  CON: n = 14 | - Ages 11-15 years - Fluent in Japanese - Secondary school - ASD diagnosis - VIQ > 70 - Motivated - No history of major mental illness^b^ - No severe behavioral problems - No hearing, visual or physical impairments |  | Adolescent:   - **TASSK** - **DSRS-C** - QPQ-A   Parent:   - **SRS-2** - **SCQ** - **VABS-2** - **CBCL** | - ***p* < .001** - ***p* < .05** - ns - ***p* < .001** - ***p* < .001** - ***p* < .001** - ***p* < .001** | - Small samples - No observational measures - No teacher ratings - No qualitative data on treatment fidelity - Lack of diagnostic assessment |
| Rabin et al. 2020 | Immediate intervention (II)  Delayed intervention (DI) | EXP 41  Delayed 42 | - Ages 12 – 17 years - ASD diagnosis - No concurrent intellectual disability - Motivated - No behavioral problems - No participation in other intervention study |  | Adolescent   - CASS - TASSK - EQ   Parents   - SSIS – Social skills - SRS-2 - CIB   Teachers   - SSIS – Social skills - SRS-2 | Both II and DI after intervention   - ***p* < .05** - ***p* < .05** - ***p* < .05** - ***p* < .05** - ***p* < .05**   n.s.  n.s. | - CASS confederate age (undergraduates) - Limited sample size parental sensitivity (only measured in Immediate intervention condition) |

*Note*. EXP = experimental group; CON = control group. The bold text means the outcome measures reach a level of significant (i.e. *p* < .001 or *p* < .05)

^a^study on the original version of the 12-week program instead the currently used 14-week program

^b^bipolar disorder, schizophrenia, or psychosis

^c^study on the 14-week program instead the currently used 16-week program

Contextual Assessment of Social Skills (CASS); Child Depression Inventory (CDI), Coding of Interactive Behavior (CIB), Empathy Quotient (EQ), Friendship Qualities Scale (FQS), Quality of Play Questionnaire (QPQ), Quality of Socialization Questionnaire (QSQ), Social Interaction Anxiety Scale (SIAS), Social Responsiveness Scale (SRS), Social Skills Rating System (SSRS), Test of Adolescent Social Skills Knowledge (TASSK), Vineland Adaptive Behavior Scales-Second Edition (VABS), Loneliness and Social Dissatisfaction Questionnaire (LSDQ), Empathy Quotient (EQ), Adaptive Behavior Assessment System (ABAS), Social Communication Questionnaires (SCQ), Vineland Adaptive Behavior Scales-Second Edition (VABS-2), Child Behavior Checklist (CBCL), Depression Self-Rating Scale for Children (DSRS-C).
