# Supplementary material for: A randomized controlled trial to examine the effectiveness of the Dutch version of the Program for the Education and Enrichment of Relational Skills (PEERS®)
Source: BMC Psychiatry. 2022 Apr 22;22:293. doi: 10.1186/s12888-022-03913-3 (PMC9034592; doi:10.1186/s12888-022-03913-3)
Supplement: Supplementary file 2 — Additional file 2. Attrition analyses outcomes. [file 12888_2022_3913_MOESM2_ESM.docx]

*Additional file 2* Attrition analyses outcomes

1. At baseline assessment (T1), seven out of 106 participants were without a CASS video recording. Reasons for not having the CASS video recording were the unavailability of a suitable confederate during the assessment (*n* = 3), technical problems (*n*=2), refusal because he/she was too anxious (*n*=2). When comparing groups of adolescents with (*n*=99) versus without (*n*=7) the primary outcome (CASS), analyses revealed no significant differences regarding sex (χ^2^ = .48, *p* = .49), TIQ (*t* (88) = .79, *p* = .44), PIQ (*t* (86) = .28, *p* = .78), VIQ (*t* (86) = -.03, *p* = .98), SRS-2 total score (*t* (103) = 1.16, *p* = .25), and ADOS-2 (*t* (72) = .60, *p* = .55). A significant difference was found for age (*t* (104) = -2.28, *p* = .03), indicating that younger participants were more inclined to have missing data on the CASS at T1 (n=7; M=13.29, SD=1.38) than participants who did have complete CASS data at T1 (n=99, M=14.65, SD=1.53).

2. No significant differences were found between the adolescents who dropped out versus those who completed the intervention on age (*t* (104) = -.30, *p* = .77), age of the parent (*t* (104) = .37, *p* = .71), sex of the adolescent (χ^2^ = .90, *p* = .34), sex of the parent (χ^2^ = .04, *p* = .83), SRS-2 total score at baseline assessment (*t* (103) = -1.70, *p* = .09), total IQ (*t* (88) = 1.53, *p* = .13), performance IQ (*t* (86) = 1.42, *p* = .16), and verbal IQ (*t* (86) = 1.50, *p* = .14).

3. *n*=26 adolescents and *n*=20 parents did complete the pre- and post-assessments (T1 and T3) but, were not able to attend the follow-up assessment (T4) due to various reasons, such as sickness, holidays, missing the email invitation, or moving to a different location. The adolescents and parents who did not attend the follow-up assessment did not statistically differ from the participants who did attend at follow-up (*n*=58 adolescents and *n*=61 parents) on all demographic characteristics, diagnostic and outcome measures as well as treatment satisfaction (all *p’s* >.05).

4. Further exploration was done into whether parents who had complete data of SRS-2 at all-time point differed significantly with parents who completed only pre- and post-assessment on treatment satisfaction. Comparing parents with only pre- and post SRS-2 data, but no follow up (*n*=20) versus parents who completed the SRS-2 at all time-points (*n*=61) showed no significant differences on adolescent (*t*(72) = 1.79, *p* = .08) and parents’ treatment satisfaction (*t*(68) = -.69, *p* = .50).
