# Supplementary material for: A randomized controlled trial to examine the effectiveness of the Dutch version of the Program for the Education and Enrichment of Relational Skills (PEERS®)
Source: BMC Psychiatry. 2022 Apr 22;22:293. doi: 10.1186/s12888-022-03913-3 (PMC9034592; doi:10.1186/s12888-022-03913-3)
Supplement: Supplementary file 3 — Additional file 3. Results Linear Mixed Model analyses; main effects of time and condition (N = 106). [file 12888_2022_3913_MOESM3_ESM.docx]

*Additional file 3* Results Linear Mixed Model analyses; main effects of time and condition (N = 106)

|  |  | **F** | **Numerator** | **Denomenator** | ***p*** |
| --- | --- | --- | --- | --- | --- |
|  |  |  | **Df** | **Df** |  |
| CASS Total | Time | 0.84 | 1 | 151.37 | .77 |
|  | Condition | 0.41 | 1 | 227.90 | .52 |
|  | Condition x Time | 1.81 | 1 | 146.13 | .18 |
| 0 Starting | Time | 4.34 | 1 | 145.19 | .04* |
|  | Condition | 4.15 | 1 | 204.42 | .04* |
|  | Condition x Time | 1.81 | 1 | 145.19 | .18 |
| 1 Total Question Asked | Time | .00 | 1 | 145.04 | .95 |
|  | Condition | .00 | 1 | 227.85 | .97 |
|  | Condition x Time | .15 | 1 | 145.04 | .70 |
| 1a Initiating Question | Time | 1.35 | 1 | 144.74 | .25 |
|  | Condition | .08 | 1 | 226.16 | .78 |
|  | Condition x Time | .03 | 1 | 144.74 | .87 |
| 1b Follow-up Question | Time | .52 | 1 | 153.78 | .47 |
|  | Condition | .10 | 1 | 221.58 | .75 |
|  | Condition x Time | .57 | 1 | 153.78 | .45 |
| 2 Topic Changes | Time | .24 | 1 | 151.81 | .63 |
|  | Condition | .55 | 1 | 214.82 | .46 |
|  | Condition x Time | .34 | 1 | 151.81 | .56 |
| 3 Vocal Expressiveness | Time | .15 | 1 | 151.51 | .70 |
|  | Condition | .04 | 1 | 217.21 | .84 |
|  | Condition x Time | 1.56 | 1 | 151.51 | .21 |
| 4 Gestures | Time | .19 | 1 | 161.72 | .66 |
|  | Condition | .17 | 1 | 209.11 | .68 |
|  | Condition x Time | 1.26 | 1 | 161.72 | .26 |
| 5 Positive Affect | Time | 4.57 | 1 | 153.55 | .03* |
|  | Condition | .03 | 1 | 221.50 | .87 |
|  | Condition x Time | .66 | 1 | 153.55 | .42 |
| 6 Kinesic Arousal | Time | .02 | 1 | 148.19 | .89 |
|  | Condition | .20 | 1 | 227.50 | .66 |
|  | Condition x Time | .25 | 1 | 148.19 | .62 |
| 7 Social Anxiety | Time | 2.05 | 1 | 155.02 | .15 |
|  | Condition | .89 | 1 | 219.19 | .35 |
|  | Condition x Time | .51 | 1 | 155.02 | .48 |
| 8 Overall Involvement | Time | 1.86 | 1 | 156.75 | .18 |
|  | Condition | 0.84 | 1 | 216.05 | .36 |
|  | Condition x Time | 1.13 | 1 | 156.75 | .29 |
| 9 Overall Quality of Rapport | Time | 3.80 | 1 | 158.29 | .05* |
|  | Condition | 0.19 | 1 | 216.81 | .66 |
|  | Condition x Time | 1.25 | 1 | 158.29 | .27 |
| 10 End Initiative | Time | 18.35 | 1 | 157.48 | <.01** |
|  | Condition | .01 | 1 | 157.48 | .92 |
|  | Condition x Time | .10 | 1 | 184.58 | .75 |
| 11 End Reason | Time | 2.85 | 1 | 172.87 | .09 |
|  | Condition | .00 | 1 | 204.61 | .99 |
|  | Condition x Time | .13 | 1 | 172.87 | .71 |
| Conversation Rating Scale Total | Time | 15.09 | 1 | 146.81 | <.01** |
|  | Condition | 4.36 | 1 | 229.82 | .04* |
|  | Condition x Time | 2.39 | 1 | 146.81 | .13 |
| Perspective taking items | Time | 9.36 | 1 | 144.01 | .00** |
|  | Condition | 0.00 | 1 | 217.90 | .99 |
|  | Condition x Time | 0.46 | 1 | 144.01 | .50 |
| SSIS-Adolescent Total | Time | 26.73 | 1 | 137.86 | .00** |
|  | Condition | 2.56 | 1 | 223.16 | .11 |
|  | Condition x Time | 5.37 | 1 | 137.86 | .02* |
| SSIS-Parent Total | Time | 29.26 | 1 | 142.17 | .00** |
|  | Condition | 2.57 | 1 | 223.99 | .11 |
|  | Condition x Time | 2.57 | 1 | 142.17 | .11 |
| SRS-2 Parent Total | Time | 59.52 | 1 | 141.72 | .00** |
|  | Condition | 2.44 | 1 | 222.98 | .12 |
|  | Condition x Time | 5.63 | 1 | 141.72 | .02* |
| SSIS-Teacher Total | Time | 2.38 | 1 | 66.59 | .13 |
|  | Condition | 1.50 | 1 | 129.00 | .22 |
|  | Condition x Time | 1.06 | 1 | 66.59 | .31 |
| SRS-2 Teacher Total | Time | 6.79 | 1 | 58.52 | .01** |
|  | Condition | 1.24 | 1 | 117.03 | .27 |
|  | Condition x Time | .30 | 1 | 58.52 | .58 |

Note. *p* ≤ .05 *, *p* ≤ .01**
